# Primary Well-Differentiated Neuroendocrine Tumor of the Kidney

**DOI:** 10.15586/jkcvhl.v10i2.277

**Published:** 2023-05-11

**Authors:** Vishnu Prasad, Charakkulam Vijay Sreelakshmi, K Ravi Chandran, Shashank Agrawal, Ginil Kumar Pooleri, Amrita Sao

**Affiliations:** Amrita Institute of Medical Sciences, Kochi, India

**Keywords:** Neuroendocrine tumor, Radical nephrectomy, Immunohistochemistry

## Abstract

Primary neuroendocrine tumors (NET) of the kidney are rare. They present with varied symptoms, making their diagnosis difficult clinically as well as pathologically. We present to you the case of a renal NET, which presented in a young female patient. A 48-year-old female patient came with an incidentally detected right renal mass during the evaluation of a nonspecific gynecological problem. She underwent contrast-enhanced computed tomography (CT) of the abdomen, which showed a 57*45*34 mm mass with enlarged retrocaval and aortocaval nodes (25*12 mm). Renal cell carcinoma was suspected as per the CT findings, and metastatic workup in the form of FDG PET CT was done in view of the unusually enlarged nodes. She underwent robot-assisted radical nephrectomy along with lymph node dissection. Surgery was uneventful, and she recovered well in the postoperative period. In the final pathology, there was confusion regarding the diagnosis, and further immunohistochemistry (IHC) was recommended by the pathologist. IHC showed synaptophysin positive, chromogranin negative, CD56 focally positive with Ki-67 of 2–3%, which was suggestive of low-grade NET of the kidney. Lymph nodes were negative. She was kept on follow-up and a Ga 68-DOTANOC scan at 3 months showed no evidence of disease. Diagnosis and management of NET of the kidney still remains a debatable and controversial topic in view of its rarity. High index of suspicion needs to be observed in patients presenting with carcinoid syndrome and a renal mass. Nuclear scans like PET scan and DOTANOC scan can accurately stage the disease. Management includes partial or radical nephrectomy depending on the tumor characteristics. Further studies are required to optimize the treatment protocols for these patients.

## Introduction

Neuroendocrine tumors (NETs) of the kidney are a rare type of cancer that arises from specialized cells called neuroendocrine cells. These tumors are characterized by their ability to produce and secrete hormones, which can lead to a range of symptoms and complications. In recent years, there has been growing interest in the diagnosis and management of NETs of the kidney, and significant progress has been made in understanding the biology and treatment of these tumors (1). We present to you a case of renal NET in a young female patient. Informed consent was taken for publication of this case.

## Case details

**Figure 1:**
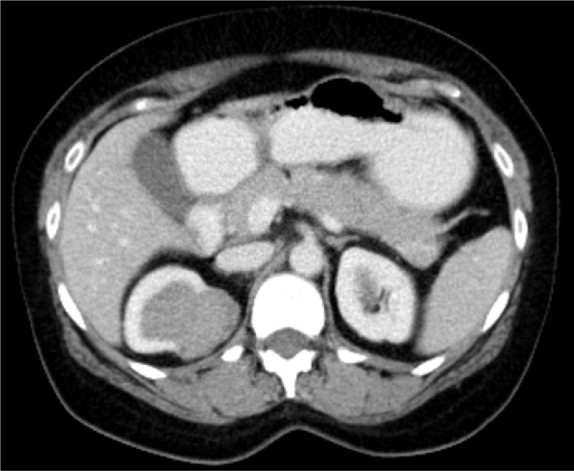
Contrast-enhanced CT scan of the abdomen showing renal mass in the right kidney.

**Figure 2:**
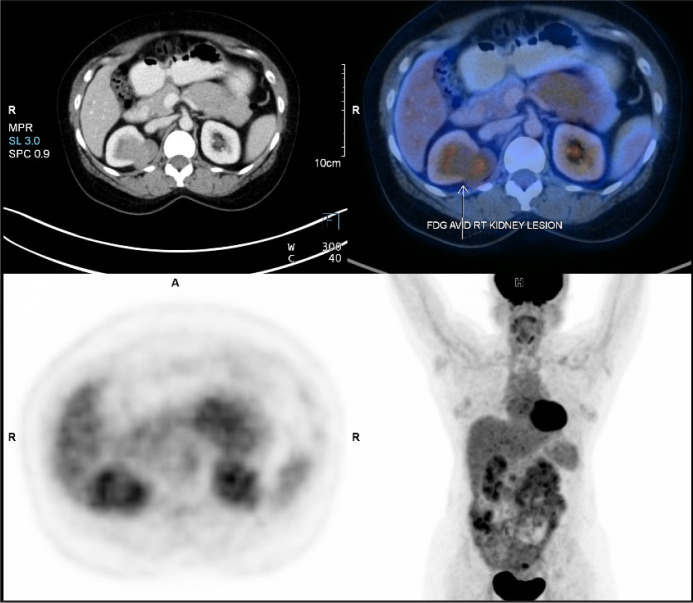
FDG PET CT showing uptake in the right renal mass.

A 48-year-old female patient came with an incidentally detected right renal mass during evaluation for menorrhagia. Ultrasound evaluation for the same revealed a right renal mass. This was further evaluated with a dedicated computed tomography (CT) scan of the abdomen, which showed a solid cystic mass lesion in the right kidney’s upper pole, which was located posteromedially and measured 57*45*34 mm with obvious enhancement. There were also multiple enlarged retrocaval and aortocaval nodes, the largest measuring 25* 12 mm (Figure 1). Her serum creatinine was 0.8 mg/dL and her hemoglobin was 11 g/dL. The uterus showed evidence of multiple fibroids. She did not have any flank pain, lower urinary tract symptoms, or hematuria. She had no comorbidities or addictions. She, however, did have a family history of breast cancer. She underwent metastatic workup by an FDG positron emission tomography (PET) CT scan (Figure 2), which showed a metabolically active heterogeneously enhancing lobulated solid cystic lesion in the right kidney along with metabolically active right renal hilar and aortocaval lymph nodes. No other areas of metastasis were found.

**Figure 3:**
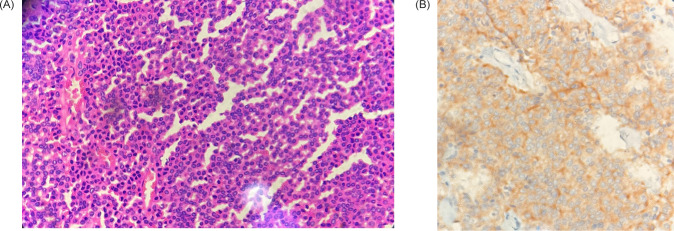
(A) Haematoxylin and Eosin staining; (B) Synaptophysin Positivity on Immunohistochemistry.

In view of these findings, a plan to do a robotic right radical nephrectomy along with lymph node dissection was made. Surgery was uneventful, and she recovered well in the postoperative period. She was discharged on postoperative day 2. The pathology report came a few days later which was suggestive of a NET of the kidney. The tumor was seen to involve the perinephric and renal sinus fat (pathological stage: pT3a). The lymph nodes were negative for tumor metastasis. Further immunohistochemistry (IHC) was advised. The tumor was found to be synaptophysin positive, chromogranin negative, and CD56 focally positive with Ki-67 of 2–3% (Figure 3). In view of the low-grade tumor and as per the discussion in the multidisciplinary tumor board, a plan to observe the patient with the required follow-up imaging was made. She underwent a Ga 68 DOTA NOC scan 3 months later, which showed no evidence of tumor. She was further followed up after a year of surgery with a CT scan of the abdomen and a chest x-ray, which revealed no evidence of local recurrence or metastasis.

## Discussion

The first case of a NET of the kidney was reported in 1966 (2) and since then, only about 200 occurrences of the disease have been recorded in medical literature, making them extremely rare (2, 3). The most frequently encountered genitourinary carcinoid tumor in females is ovarian carcinoid (92%) followed by renal carcinoid tumors (5%). Patients over the age of 40 with tumors more than 4 cm in size are at relatively greater risk of metastases.

The common symptoms presented by patients other than the usual features of carcinoid syndrome are abdominal or flank pain followed usually by hematuria, constipation, weight loss, and urinary tract infections (4). Incidentally, detected tumors like the one we presented, account for one-fourth of the cases.

According to a study by Krishnan et al., individuals with horseshoe kidney have around 62 times higher risk of developing NETs (5). Though the pathogenesis of carcinoid tumors of kidney has been debatable, el-Naggar et al. found a loss of heterozygosity at one locus on chromosome 3p21 and hypothesized that this aberration, which is common in renal cell carcinoma (RCC), is a precursory event shared by all renal neoplasms, including carcinoid tumors (6).

The usual CT presentation of a carcinoid tumor is a well-circumscribed, nonenhancing, or slightly enhancing mass with a solid component, but in some cases cystic components or calcifications are also seen (1, 7). According to Henry et al., in which seven cases were reviewed, 71.4% showed calcification (7). Our patient’s CT scan showed a heterogeneously enhancing lobulated solid cystic lesion with multiple regional lymph nodes.

Fourteen per cent of all renal NETs are initially misdiagnosed after histopathological investigation as conventional RCC, papillary RCC, papillary transitional cell carcinoma, atypical adenocarcinoma, or Wilms tumor (8). Neuroendocrine carcinoma rarely expresses CD10, while at least one neuroendocrine marker such as synaptophysin, chromogranin, or CD56 is usually expressed. The diagnostic stains for renal carcinoid are chromogranin A and the more sensitive synaptophysin. NET phenotype is supported by CD 56 positivity (9–11). RCC and urothelial cancer, in contrast, frequently express CD10 but not the neuroendocrine markers mentioned above. Therefore, to diagnose renal NET accurately, a combination of investigations for neuroendocrine markers and CD10 will be necessary (10, 11). A clinicopathological study of 21 cases done by Hansel et al. showed in their study that immunostains were frequently positive for synaptophysin (n = 18/20), chromogranin (n = 13/20), Cam5.2 (n = 14/16), and vimentin (n = 12/15) (12). The IHC of the present case was synaptophysin positive, CD56 focally positive, but chromogranin negative. For NETs, Ki67 is a reliable pathological grading marker (13); the present subject showed Ki67 of 2–3% of proliferation index, implying that the patient has grade G1 NET according to the European Neuroendocrine Tumor Society (ENETS) guidelines and the 2010 World Health Organization (WHO) classification (13).

The most widely used functional imaging technique for cancer detection is fluorodeoxyglucose-PET (FDG-PET). For well-differentiated NETs, In-pentetreotide was more sensitive than FDG-PET, whereas it showed greater sensitivity for poorly differentiated NETs (14). In the review by Andreas et al., FDG PET was found to be even more reliable than KI65 classification (15). FDG-PET is frequently employed to assess tumor metabolism in cancer patients in different stages of the disease (16).

Based on the location, diameter, and type of renal carcinoid tumors, the gold standard treatment for these tumors is nephrectomy, either partial or radical (17). Procedure development has seen significant advances, particularly in robotic surgery, and robot- assisted partial nephrectomy has successfully treated cT1 renal tumors and has had positive perioperative and oncologic outcomes (18). If any enlarged nodes are seen on scans, regional lymph node dissection should be performed. When the renal mass is tiny, or when the mass is close to the ureter or another nearby structure, nephron-sparing surgery has the advantage of maintaining renal function (17).

68Ga-labeled PET CT has been found to be superior to other imaging modalities for NETs and is a helpful technique for diagnosis, staging, and monitoring following treatment for recurrence or metastasis of carcinoid tumors (19, 20). Our patient underwent a Ga 68 DOTA NOC scan 3 months later, which showed no evidence of the tumor. She is currently under further follow-up.

Teegavarapu PS et al. reported six cases of small cell carcinoma of the kidney. The mean age of diagnosis was 65 years. Surgery and chemotherapy were the mainstays of treatment. They reported a median survival of 17.3 months (21).

## Conclusion

Little is understood about the pathogenesis, clinical features, and management of NETs of the kidney. Literature is limited to only a few case studies. More information is required to optimize the diagnosis and treatment plan for such patients.
